# Long-term oncologic outcomes of unselected triple-negative breast cancer patients according to *BRCA1/2* mutations

**DOI:** 10.1038/s41698-024-00559-0

**Published:** 2024-04-30

**Authors:** Woong Ki Park, Soo Yeon Chung, You Jin Jung, Changhee Ha, Jong-Won Kim, Seok Jin Nam, Seok Won Kim, Jonghan Yu, Byung Joo Chae, Jeong Eon Lee, Sung-Won Kim, Jai Min Ryu, Korean Hereditary Breast Cancer Study Group

**Affiliations:** 1 Division of Breast Surgery, Department of Surgery, Samsung Medical Center, Sungkyunkwan University School of Medicine, Seoul, South Korea; 2 Sungkyunkwan University School of Medicine, Seoul, South Korea; 3 Department of Laboratory Medicine and Genetics, Samsung Medical Center, Sungkyunkwan University School of Medicine, Seoul, South Korea; 4 Department of Surgery, The Breast Care Center, Daerim St. Mary’s Hospital, Seoul, South Korea

**Keywords:** Breast cancer, Surgical oncology

## Abstract

Triple-negative breast cancer (TNBC) patients are more likely to have *BRCA1/2* mutations, with a prevalence rate of about 10–20%. Although several studies have analyzed the oncologic outcomes between *BRCA1/2* carriers and non-carriers, the impact on breast cancer patients is still unclear. A retrospective review was performed to determine the long-term outcomes of TNBC patients, focusing on the impact of *BRCA1/2* mutations. A total of 953 TNBC patients who underwent primary breast cancer surgery from June 2008 to January 2016 were included. We examined long-term outcomes, including contralateral breast cancer (CBC) incidence, recurrence patterns, and survival rates over a median follow-up of 80.9 months (range 3–152 months). 122 patients (12.8%) had *BRCA1/2* mutations. *BRCA1/2* mutation carriers were significantly younger at diagnosis and more likely to have a family history of breast/ovarian cancer. CBC incidence at 60, 120, and 150 months was significantly higher in *BRCA1/2* mutation carriers compared to non-carriers (*P* = 0.0250, 0.0063, and 0.0184, respectively). However, there were no significant differences in disease-free survival, overall survival, breast cancer-specific survival, or distant-metastasis-free survival between the two groups. *BRCA1/2* mutation status was a significant risk factor for CBC (HR = 6.242, *P* < 0.0001). Interestingly, among 29 patients with CBC recurrence, 24 patients (82.8%) had recurring TNBC subtype and among the CBC recurrence patients, 19 patients (65.5%) resumed chemotherapy. In the TNBC subtype, appropriate genetic testing and counseling are pivotal for surgical decisions like risk-reducing mastectomy (RRM). Furthermore, long-term surveillance is warranted, especially in *BRCA1/2* carriers who did not receive RRM.

## Introduction

Breast cancer comprises subtypes with distinct morphologies and clinical implications. Triple-negative breast cancer (TNBC) is defined by little or lack of expression of estrogen receptor (ER), progesterone receptor (PR), and human epidermal growth factor receptor 2 (HER-2)^1^. The TNBC subtype has poor oncologic outcomes; a higher risk of early recurrence and a lower risk of late recurrence compared to luminal-type breast cancer^2–4^. Among all breast cancer patients, TNBC patients are more likely to have *BRCA1/2* germline mutations, with a prevalence rate of approximately 10–20%^5–8^.

Several studies have analyzed prognosis and oncologic outcomes between *BRCA1/2* carriers and non-carriers, but whether *BRCA1/2* carriers have a worse prognosis is unclear. Some reports have shown that *BRCA1/2* mutation carriers have poor overall survival (OS) relative to non-carriers^9,10^. Other studies have reported no difference in OS between *BRCA1/2* carriers and non-carriers^11–14^, and some reports indicate that *BRCA1/2* carriers showed better survival outcomes than non-carriers^6^. Recently, results from the OlympiA trial reported improved survival with the use of poly (adenosine diphosphate-ribose) polymerase inhibitor (PARPi), olaparib in *BRCA1/2* mutation associated early breast cancer patients^15^. To date, only a few studies have focused on the effect of *BRCA1/2* mutations on long-term oncologic prognosis in unselected TNBC patients.

Previously, we reported the prevalence and oncologic outcomes (median follow-up of 53.6 months) of germline *BRCA1/2* mutations among unselected Korean patients with TNBC. Poor contralateral breast cancer (CBC)-free survival and a prevalence of 14.5% in TNBC patients aged ≤ 60 years were observed, contributing to the update in *BRCA1/2* genetic testing guidelines in Korea^16^. Here, we present the follow-up results of our previous study, which assessed the long-term oncologic outcomes and recurrence rates after 5 years with a median follow-up of 80.9 months.

## Results

### Baseline characteristics

The baseline patient characteristics according to *BRCA1/2* mutation are shown in Table 1. All patients were Korean. *BRCA1/2* mutation carriers were significantly younger (mean age 45.5 ± 11.0 vs. 50.1 ± 10.8) and premenopausal (63.1% vs. 47.3%) at the time of diagnosis compared to non-carriers (*P* = 0.002 and *P* = 0.0011, respectively). Also, *BRCA1/2* mutation carriers were more likely to have a family history of breast cancer and/or ovarian cancer (40.2% vs. 9.4%, *P* < 0.0001), personal history of ovarian cancer (9.0% vs. 0.5%, *P* < 0.0001), bilateral breast cancer (4.9% vs. 1.2%, *P* = 0.0105), and a higher nuclear grade (86.0% vs. 73.4%, *P* = 0.0140). Pathologic stage, lympho-vascular invasion, multiplicity, and proportion of mastectomy were not significantly different in those with or without *BRCA1/2* mutation. Although differences in the proportion of adjuvant chemotherapy (81.2% vs. 70.2%, *P* = 0.0120) and neo-adjuvant chemotherapy (13.1% vs. 21.8%, *P* = 0.0273) were observed, the total proportion of chemotherapy treatment was comparable between the two groups (94.3% vs. 89.4%, *P* = 0.1314).

**Table 1:** Patient characteristics

	*BRCA1/2*(+), *n* (%)	*BRCA1/2* (−), *n* (%)	*P*-value
Number	122 (12.8)	831 (87.2)	
Age, mean ± SD	45.5 ± 11.0	50.1 ± 10.8	
*Age at diagnosis*			0.002
<40	36 (29.5)	147 (17.7)	
≥40	86 (70.5)	684 (82.3)	
*Menopausal status*			0.0011
Postmenopausal	45 (36.9)	438 (52.7)	
Premenopausal	77 (63.1)	393 (47.3)	
*Bilateral BC*			0.0105
Yes	6 (4.9)	10 (1.2)	
No	116 (95.1)	821 (98.8)	
*Personal history of OC*			<0.0001
Yes	11 (9.0)	4 (0.5)	
No	111 (91.0)	827 (99.5)	
*Family history of BC and/or OC*			<0.0001
Yes	49 (40.2)	78 (9.4)	
No	73 (59.8)	753 (90.6)	
*Histology*			0.1182
IDC	105 (86.1)	727 (87.5)	
ILC	0	7 (0.8)	
DCIS	1 (0.8)	29 (3.5)	
Others	16 (13.1)	68 (8.2)	
*pT stage*			0.4614
pT0	1 (0.8)	32 (3.9)	
pT1	56 (46.0)	356 (42.8)	
pT2	52 (42.6)	355 (42.7)	
pT3	7 (5.7)	32 (3.9)	
pT4	0	2 (0.2)	
Complete remission after NAC	6 (4.9)	51 (6.1)	
Unknown	0	3 (0.4)	
*pN1 stage*			0.6633
pN0	81 (66.4)	565 (68.0)	
pN1	31 (25.4)	176 (21.2)	
pN2	6 (4.9)	44 (5.3)	
pN3	4 (3.3)	36 (4.3)	
No operation	0	10 (1.2)	
*Nuclear grade*			0.014
Low	1 (0.9)	11 (1.3)	
Intermediate	11 (9.0)	160 (19.3)	
High	105 (86.0)	610 (73.4)	
Unknown	5 (4.1)	50 (6.0)	
*LVI*			0.7551
Yes	34 (27.9)	243 (29.2)	
No	88 (72.1)	588 (70.8)	
*Multiplicity*			0.5968
Yes	16 (13.1)	122 (14.9)	
No	106 (86.9)	695 (85.1)	
*Breast surgery*			0.3348
BCS	98 (80.3)	634 (76.4)	
TM	24 (19.7)	196 (23.6)	
*Axillary surgery*			0.1794
SLNB	77 (63.1)	465 (56.0)	
ALND	45 (36.9)	355 (42.7)	
Not done	0	11 (1.3)	
*Adjuvant radiotherapy*			0.6255
Yes	105 (86.1)	701 (84.4)	
No	17 (13.9)	130 (15.6)	
*Chemotherapy (Adjuvant chemotherapy + NAC)*			0.1314
Yes	115 (94.3)	743 (89.4)	
No	7 (5.7)	88 (10.6)	
*Adjuvant chemotherapy*			0.012
Yes	99 (81.1)	583 (70.2)	
No	23 (18.9)	248 (29.8)	
*NAC*			0.0273
Yes	16 (13.1)	181 (21.8)	
No	106 (86.9)	650 (78.2)	
*Chemotherapy regimen*			0.9898
AC	11 (9.6)	69 (9.3)	
AC+T	30 (26.1)	175 (23.5)	
FAC	45 (39.1)	272 (36.6)	
Others	9 (7.8)	48 (6.5)	
Unknown	20 (17.4)	179 (24.1)	
*Recurrence*			0.2483
Yes	31 (25.4)	173 (20.8)	
No	91 (74.6)	658 (79.2)	
*Distant metastasis*			0.2739
Yes	13 (10.7)	119 (14.3)	
No	109 (89.3)	712 (85.7)	
*Expire*			0.4796
Yes	18 (14.8)	144 (17.3)	
No	104 (85.2)	687 (82.7)	
*Distant metastasis site*			0.2182
Bone	0	10 (8.4)	
Lung	4 (30.8)	13 (10.9)	
Brain	2 (15.4)	17 (14.3)	
Liver	0	1 (0.8)	
Multiple	6 (46.1)	66 (55.5)	
LNs	1 (7.7)	12 (10.1)	

*SD* standard deviation, *BC* breast cancer, *OC* ovarian cancer, *IDC* invasive ductal carcinoma, *ILC* invasive lobular carcinoma, *DCIS* ductal carcinoma in situ, *NAC* neo-adjuvant chemotherapy, *LVI* lymphovascular invasion, *BCS* breast-conserving surgery, *TM* total mastectomy, *SLNB* sentinel lymph node biopsy, *ALND* axillary lymph node dissection, *AC* adriamycin, cyclophosphamide, *T* paclitaxel, *FAC* fluorouracil, adriamycin and cyclophosphamide.

### Long-term oncologic outcomes

**Fig. 1:**
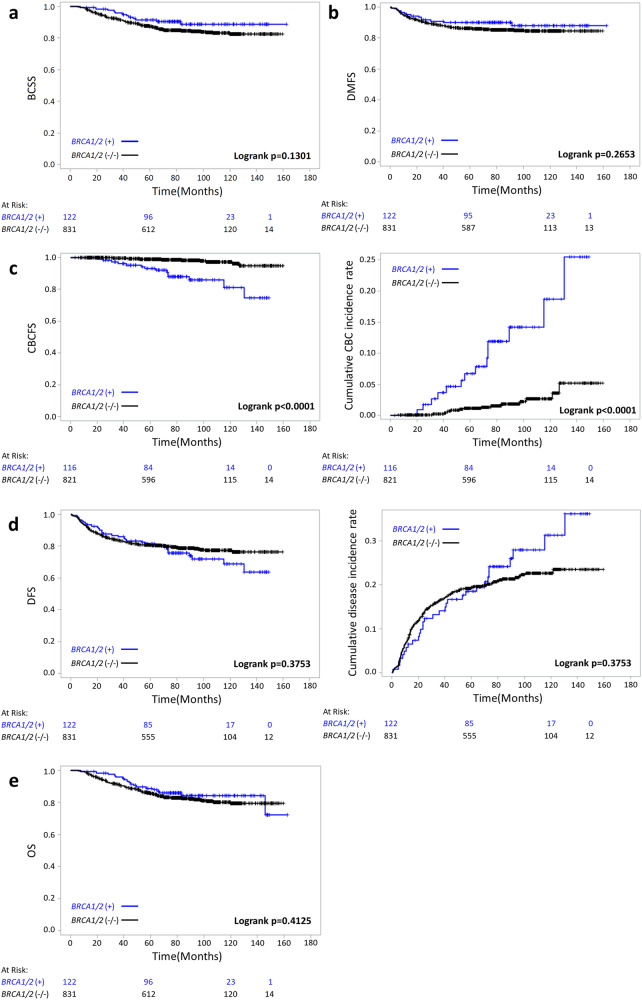
Kaplan–Meier curves. Kaplan–Meier curves with corresponding log-rank tests for BCSS (**a**), DMFS (**b**), CBCFS and cumulative CBC incidence rate (**c**), DFS and cumulative disease incidence rate (**d**), and OS (**e**) according to BRCA1/2 mutation status. DFS disease-free survival, DMFS distant metastasis-free survival, CBCFS contralateral breast cancer-free survival, OS overall survival, BCSS breast cancer-specific survival.

With a median follow-up duration of 80.9 months (range, 3–152 months), we compared the long-term oncologic outcomes between *BRCA1/2* mutation carriers and non-carriers including the cumulative risks on 60-, 120-, and 150-month period (Table 2 and Fig. 1). 60- and 120-month cumulative disease incidence rates were 18.5% and 31.2% for *BRCA1/2* carriers vs. 19.3% and 22.7% for non-carriers (*P* = 0.8346 and 0.1359, respectively). Although not statistically significant, the cumulative disease incidence rate at 150 months was higher in *BRCA1/2* mutation carriers compared to non-carriers (36.2% vs. 23.5%, *P* = 0.0800). 60-, 120-, and 150-month CBC incidence rate estimates were 6.7%, 18.7%, and 25.5% for *BRCA1/2* mutation carriers vs. 1.1%, 2.7%, and 5.2% for non-carriers, which showed statistically significant differences (*P* = 0.0250, 0.0063, and 0.0184, respectively). There were no significant differences in cumulative mortality rates between the two groups in all three time periods. 60-, 120-, and 150-month breast cancer-specific survival (BCSS) had no significant difference, but poorer BCSS was observed in the non-carrier group (91.4%, 88.8%, and 88.8% for *BRCA1/2* carriers vs. 87.4%, 82.4%, and 82.4% for non-carriers *P* = 0.1571, 0.0724, and 0.0724, respectively). There was no significant difference in distant-metastasis free survival (DMFS) between *BRCA1/2* mutation carriers and non-carriers (*P* = 0.2254, 0.3302, and 0.3302, respectively).

**Table 2:** Oncologic outcomes

	Months	*BRCA1/2* (+) (%)	*BRCA1/2* (−) (%)	*P*-value*
DFS (cumulative risk)	60	81.5 (18.5)	80.7 (19.3)	0.8346
	120	68.8 (31.2)	77.3 (22.7)	0.1359
	150	63.8 (36.2)	76.5 (23.5)	0.0800
DMFS (cumulative risk)	60	90 (10)	86.4 (13.6)	0.2254
	120	88 (12)	84.5 (15.5)	0.3302
	150	88 (12)	84.5 (15.5)	0.3302
CBCFS (cumulative risk)	60	93.3 (6.7)	98.9 (1.1)	0.0250
	120	81.3 (18.7)	97.3 (2.7)	0.0063
	150	74.5 (25.5)	94.8 (5.2)	0.0184
OS (cumulative risk)	60	88.8 (11.2)	85.7 (14.3)	0.3192
	120	84.4 (15.6)	79.3 (20.7)	0.2020
	150	72.3 (27.7)	79.3 (20.7)	0.5490
BCSS (cumulative risk)	60	91.4 (8.6)	87.4 (12.6)	0.1571
	120	88.8 (11.2)	82.4 (17.6)	0.0724
	150	88.8 (11.2)	82.4 (17.6)	0.0724

*DFS* disease-free survival, *DMFS* distant metastasis-free survival, *CBCFS* contralateral breast cancer-free survival, *OS* overall survival, *BCSS* breast cancer-specific survival.

*P-values of the log-rank test were used.

### Recurrence patterns according to *BRCA1/2* mutation status

In *BRCA1/2* mutation carriers, there were 6 locoregional recurrences, 12 distant metastases, and 7 CBC recurrences within five years. Late recurrence patterns in *BRCA1/2* mutation carriers who had no recurrence within the first 5 years showed 1 locoregional recurrence, 1 distant metastasis, and 7 CBC recurrences (Tables 3 and 4). Early and late locoregional recurrence and distant metastasis showed no significant differences between *BRCA1/2* mutation carriers and non-carriers (*P* = 0.1409, 0.4878, and 0.2754, 0.8327, respectively). Notably, the CBC recurrence rate showed a significant difference between *BRCA1/2* mutation carriers and non-carriers in both early- and late recurrences (*P* = 0.0002 and 0.0001, respectively). A total of 15 ovarian cancer occurrences were observed in the total population. 11 cases were observed in the *BRCA1/2* mutation carrier group and 4 cases were observed in the non-carrier group.

**Table 3:** Recurrence within 5 years after primary treatment by *BRCA1/2* mutations

Early recurrence		*BRCA1/2* (+)	*BRCA1/2* (−)	*P*-value*
		*n* (%)	*n* (%)	
Locoregional recurrence	Yes	6 (27.27)	73 (46.79)	0.1409
	No	16 (72.73)	83 (53.21)	
Distant metastasis	Yes	12 (54.55)	111 (71.15)	0.2754
	No	10 (45.45)	45 (28.85)	
Distant metastasis site	Bone	0	9 (8.11)	0.225
	Lung	4 (33.33)	10 (9.01)	
	Brain	2 (16.67)	17 (15.32)	
	Liver	0	1 (0.91)	
	Multiple	6 (50.00)	63 (56.76)	
	LNs	0	11 (9.91)	
Contralateral breast cancer recurrence	Yes	7 (31.82)	8 (5.13)	0.0002
	No	15 (68.18)	148 (94.87)	

For comparison of distant metastasis site, Fisher’s exact test was used.

*P-values of the log-rank test were used for locoregional recurrence, distant metastasis, and contralateral breast cancer recurrence.

**Table 4:** Recurrence after 5 years after primary treatment by *BRCA1/2* mutations

Late recurrence		*BRCA1/2* (+)	*BRCA1/2* (−)	*P*-value*
		*n* (%)	*n* (%)	
Locoregional recurrence	Yes	1 (11.11)	3 (17.65)	0.4878
	No	8 (88.89)	14 (82.35)	
Distant metastasis	Yes	1 (11.11)	8 (47.06)	0.8327
	No	8 (88.89)	9 (52.94)	
Distant metastasis site	Bone	0	1 (12.50)	0.333
	Lung	0	3 (37.50)	
	Multiple	0	3 (37.50)	
	LNs	1 (100)	1 (12.50)	
Contralateral breast cancer recurrence	Yes	7 (77.78)	7 (41.18)	0.0001
	No	2 (22.22)	10 (58.82)	

For comparison of distant metastasis sites, Fisher’s exact test was used.

*P-values of the log-rank test were used for locoregional recurrence, distant metastasis, and contralateral breast cancer recurrence.

Among 29 patients with CBC recurrence, 24 patients (82.8%) had TNBC breast cancer again and among recurred patients, 19 patients (65.5%) resumed chemotherapy. Overall, 14 patients were *BRCA1/2* mutation carriers, and 15 patients were non-carriers. Among 14 *BRCA1/2* mutation carriers with CBC recurrence, 11 patients (78.6%) had recurred triple-negative type breast cancer and 10 patients (71.4%) resumed chemotherapy. Additionally, within the 14 *BRCA1/2* mutation carriers with CBC recurrence, 12 patients had *BRCA1* mutation, and 2 patients had *BRCA2* mutation.

### Risk factors of CBC occurrence

To investigate the risk factors of CBC, we performed univariate and multivariate analyses using the Cox regression model (supplementary Table S1). Age at diagnosis was classified into 2 groups with a cutoff value of 40 years. Although younger age had a higher risk of CBC events, there was no significance (HR = 2.011, *P* = 0.074 in the univariate model and HR = 1.663, *P* = 0.200 in the multivariate model). TNM stage was not a significant risk factor. Notably, *BRCA1/2* mutation status was a meaningful risk factor for CBC events according to univariate analysis (HR = 6.580, *P* < 0.0001) and multivariate analysis (HR = 6.242, *P* < 0.0001).

## Discussion

In our previous study, with a median follow-up period of 53.6 months, we demonstrated the high prevalence of *BRCA1/2* mutation in unselected Korean TNBC patients and the higher incidence rate of CBC in *BRCA1/2* mutation carriers compared to non-carriers^16^. With a median follow-up duration of 80.9 months, this long-term follow-up study showed a consistently increased CBC incidence rate in *BRCA1/2* mutation carriers compared to non-carriers. There were no significant differences in disease-free survival (DFS), OS, BCSS, and DMFS between the two groups. Additionally, *BRCA1/2* mutation was a significant risk factor for CBC occurrence with an HR of 6.242. Among patients with CBC recurrence, ~80% had TNBC-type as recurred CBC, and over 60% resumed chemotherapy.

We observed significantly younger age of onset and higher tumor nuclear grade for *BRCA1/2* mutation carriers compared to non-carriers. In a study of 774 TNBC patients, Wong-Brown et al. also reported similar characteristics of younger age of onset for *BRCA1* mutation carriers within their TNBC study group^8^. Brekelmans et al. demonstrated that *BRCA1* mutation carriers had more histologic grade III tumors compared to sporadic breast cancer patients^10^. These characteristics of *BRCA1/2* mutation carriers may have been one of the factors affecting DFS, although not statistically significant, lower 150-month DFS was observed (63.8% for *BRCA1/2* mutation carrier group vs. 76.5% for non-carrier group).

There have been many studies comparing the prognosis of *BRCA1/2* mutation carriers to non-carriers. The common point of these study’s results was that there were no significant differences in survival according to *BRCA1/2* mutation status^17,18^. This was consistent in our study as well, demonstrating no significant differences in DFS, OS, and BCSS between *BRCA1/2* mutation carriers and non-carriers. Notably, we observed good survival rates at 10 years in both groups (88% vs. 84.5% DMFS, 88.8% vs. 82.4% BCSS and 84.4% vs. 79.3% OS). The Prospective Outcomes in Sporadic versus Hereditary breast cancer (POSH) study reported the 10-year overall survival of 558 TNBC patients with or without *BRCA1/2* mutations. In this prospective study, TNBC patients with *BRCA* mutation showed a 10-year overall survival of 72% and TNBC patients without *BRCA* mutation showed 69% 10-year overall survival^18^.

A prospective cohort study including 2213 *BRCA1/2* mutation carriers eligible for CBC analysis revealed that CBC risk was lower when the first breast cancer was diagnosed at age 40 years or higher^19^. We analyzed *BRCA1/2* mutation carriers with a cut-off age of 40 and 50 years to investigate the differences in CBC incidence. Both age cut-offs revealed no significant difference in CBC incidence (*P* = 0.9023 and 0.6199, respectively). Giannakeas et al. reported the annual risk and 25-year cumulative risk of CBC using the surveillance, epidemiology, and end results (SEER) database. With an annual risk of ~0.4% per year, the cumulative CBC risk at 12.5 years was 4.6% in invasive breast cancer patients^20^. Although our study cohort only included TNBC patients, the cumulative CBC risk was comparable in the *BRCA1/2* mutation non-carrier group (5.2% at 12.5 years). However, cumulative CBC risk in *BRCA1/2* mutation carriers was 25.5% at 12.5 years. In another study including 223 breast cancer patients with *BRCA1* mutation, CBC incidence was higher in the *BRCA1* mutation group compared to sporadic breast cancer patients^10^. This result supports our study’s findings. Additionally, we revealed that *BRCA1/2* mutation status was a significant risk factor for CBC incidence in a multivariate analysis.

The aggressive characteristics of TNBC are well reviewed in previous articles^1,11^. In a study that evaluated the recurrence patterns and outcomes of early-stage breast cancer, 310 TNBC patients were included. These patients had a higher risk of breast cancer-free interval events in the first 4 years after diagnosis^3^. Our study also investigated the recurrence patterns of TNBC including early- and late-recurrences. Although the majority of events occurred within the first 5 years, 13% of all recurrence events were observed after 5 years of initial diagnosis. Especially, CBC event rate was consistently higher in the *BRCA1/2* mutation carriers in both early-, and late recurrences. Therefore, long-term active surveillance may be beneficial in the early detection of recurrences or distant metastasis.

There have been several reports regarding the impact of *BRCA1/2* mutation results on decision-making for risk-reducing mastectomy (RRM). Chiba et al. reported an 82.5% rate of RRM in patients with known *BRCA* mutation prior to surgery compared to 29% in those unaware of the *BRCA* mutation status at the time of surgery^21^. Similar rates were shown in a study of 220 *BRCA* mutation-associated breast cancer patients (76.4% in the group aware of their *BRCA* mutation vs. 14.7% in the group unaware of their *BRCA* mutation)^22^. These factors were also compared in a study of 344 *BRCA* mutation breast cancer patients, which was conducted in our institution. The results demonstrated a 45% rate of RRM in the group aware of their *BRCA* mutation vs. 2% in the group unaware of their *BRCA* mutation^23^. Our study revealed that *BRCA1/2* mutation carriers have an increased risk of CBC occurrence and will require another systemic chemotherapy. Therefore, confirming the *BRCA1/2* mutation status prior to surgery is vital.

In Korea, *BRCA1/2* mutation testing is performed based on the Korean clinical practice guidelines for breast cancer. Our study included patients included TNBC patients from 2008 to 2016. At that time, the 6th clinical practice guideline was applied, in which TNBC patients were not candidates for *BRCA1/2* mutation testing^24^. The low rate of mastectomy observed in our study’s cohort can be explained by this fact. The Korean health insurance policy was updated in July 2020, to include TNBC patients aged 60 or under for *BRCA1/2* mutation testing, and the currently applied 10^th^ Korean clinical practice guideline for breast cancer has identical *BRCA1/2* mutation testing recommendations for TNBC patients^25^.

To the best of our knowledge, this report is the first single large institution study to analyze the long-term outcomes of unselected TNBC patients according to *BRCA1/2* mutation. Furthermore, we investigated the recurrence patterns including early- and late-recurrence patterns. We hope our results may help in surgical decision-making and surveillance strategies.

Our study does have limitations. Since it was a non-randomized retrospective study, it included incomplete patient data, which introduces the possibility of other confounding variables, whether measured or unmeasured. The study included patients from 2008 to 2016; a period during which treatment guidelines and adjuvant treatments evolved, potentially affecting the results. Additionally, since the collected data is relatively old, there may be unclear or missing information that may have affected the results. *BRCA1/2* mutation was retrospectively tested in our study. Although the cohort was ‘unselected’ for age or family history, only patients with available biobank tissue were included, making potential for unintentional selection bias. Additionally, *BRCA1/2* mutation testing was performed using the Sanger and NGS methods in our study. These methods have limitations in detecting large deletions/duplications or copy number variations (CNVs). In such cases, multiplex ligation-dependent probe amplification (MLPA) should be performed. However, since the Korean population has a lower frequency of CNVs and due to the Korean insurance policy of testing *BRCA1/2* mutation^26,27^, our testing methods still have an acceptable value.

In conclusion, our study identified that *BRCA1/2* mutation was a significant risk factor for CBC occurrence in TNBC patients. Notably, approximately 80% of patients experiencing CBC were again diagnosed with the TNBC subtype, and over 60% underwent chemotherapy following recurrence. These findings highlight the critical importance of conducting *BRCA1/2* genetic testing prior to initial surgical intervention and engaging in thorough discussions with patients regarding the option of contralateral RRM.

Moreover, our analysis revealed that the event rate for CBC remains significantly elevated beyond the 5-year mark, particularly among *BRCA1/2* mutation carriers. This observation highlights the necessity for prolonged surveillance of TNBC patients with *BRCA1/2* mutations, especially those who have not received RRM. Implementing such measures may potentially improve patient outcomes by facilitating early detection and intervention.

## Methods

### Study population

**Fig. 2:**
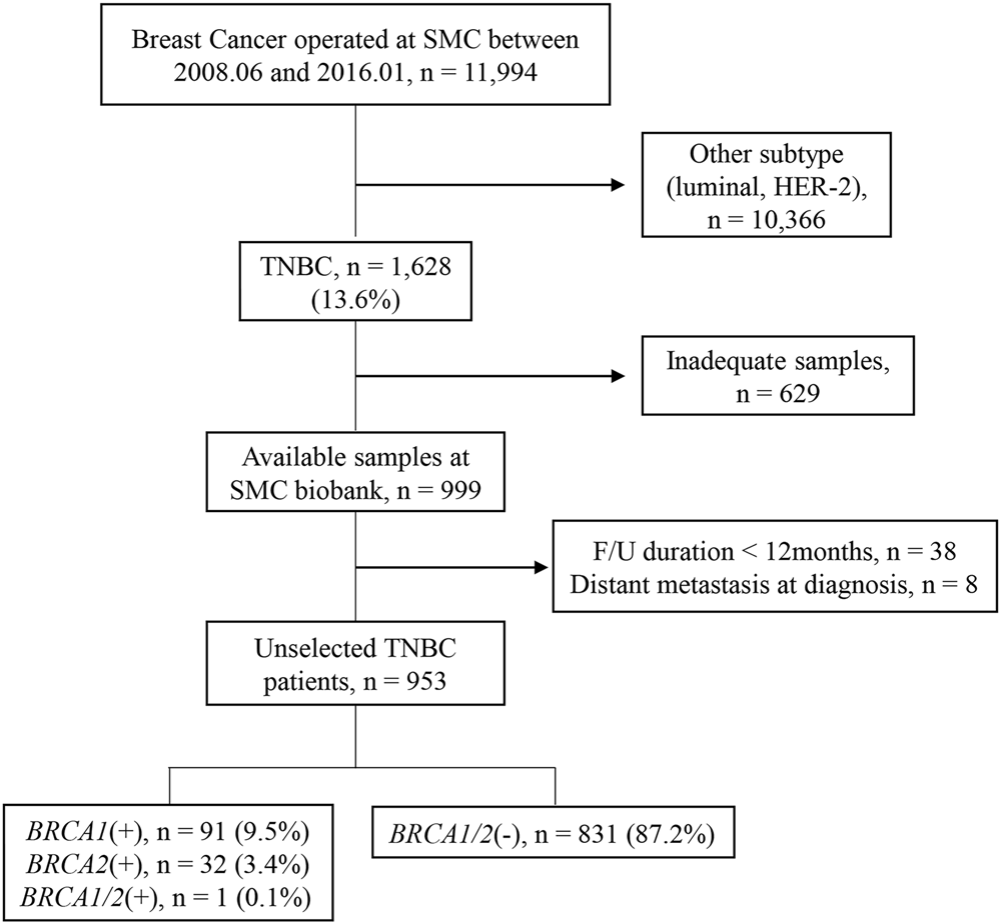
Schematic diagram of patient selection. SMC Samsung Medical Center, TNBC triple-negative breast cancer.

A schematic diagram of the study population is shown in Fig. 2. The details of the study population have been described in our previous study^16^. Briefly, unselected TNBC patients with samples available for *BRCA1/2* genetic testing were included for analysis. Next-generation DNA sequencing (NGS) method was used for genetic testing. Within this cohort, 103 patients, who met the criteria for Korean genetic screening at that time, had been examined by the Sanger method. ‘Unselected’ patients are referred to as patients not selected for age at diagnosis of a family history of breast and/or ovarian cancer.

In this follow-up study, patients with a follow-up duration of less than 12 months, and patients with distant metastasis at initial presentation were excluded from analysis. Patients who deceased within 12 months after the first diagnosis were included in the analysis. Overall, the remaining 953 patients were included in this study. 91 patients (9.5%) were *BRCA1* mutation carriers, 32 patients (3.4%) were *BRCA2* mutation carriers, and one patient had both *BRCA1/2* mutations. Long-term oncologic outcomes including CBC incidence, recurrence patterns, and survival according to *BRCA1/2* mutation status were analyzed.

### Data collection

We collected the following clinical information: *BRCA1/2* genetic test results, age at breast and/or ovarian cancer diagnosis, menopausal status, and family history of breast cancer and/or ovarian cancer. We collected the following pathologic information: diagnosis, tumor size, lymph node (LN) metastasis (number of LNs in metastasis), hormone receptor status (ER, PR), HER-2 status, histologic grade, nuclear grade, and pathologic stage according to the 7th edition of the American Joint Committee on Cancer classification. We collected the following treatment information: information on surgery, date of operation(s), adjuvant chemotherapy, neo-adjuvant chemotherapy, adjuvant radiation therapy, and/or anti-hormone therapy.

Staining of 1% of cells or more was considered positive for ER and PR^28^. Only membrane staining intensity and pattern were evaluated using the recommendations of the 2013 American Society of Clinical Oncology (ASCO)/College of American Pathologists (CAP)^29^. TNBC was immunohistochemically defined as ER/PR-negative and lacking overexpression of HER-2. Recurrence or metastasis status, as well as survival data (including the date of occurrence), were also collected. DFS was defined as the interval from the date of diagnosis to recurrence, distant metastasis, or death or censored at the last follow-up date. CBCFS was defined as the interval from the date of diagnosis to CBC occurrence of censored at the last follow-up date. OS was defined as the interval from the date of diagnosis to death from any cause or censored at the last follow-up date. DMFS was defined as the interval from the date of diagnosis to distant metastasis or censored at the last follow-up date. BCSS was defined as the interval from the date of diagnosis to death caused by breast cancer progression or censored at the last follow-up date. Recurrence patterns (locoregional recurrence, distant metastasis, CBC incidence) were classified into early- and late recurrences based on a 5-year time frame.

### Statistical analysis

The patient characteristics were compared using an independent sample *t*-test for continuous variables and Chi-square or Fisher’s exact test for categorical variables. DFS, OS, BCSS, and CBCFS were estimated using the Kaplan–Meier method, and the *P*-values of the log-rank test were used to assess significance. Univariate and multivariate analyses were performed using the Cox regression model. Variables that were considered relevant or showed a *P*-value < 0.05 were included in the multivariate Cox regression model. All *P*-values were two-sided and a *P*-value < 0.05 was considered statistically significant in our study. All statistical analyses were conducted using SAS version 9.4 (SAS Institute, Cary, NC, USA) and R3.4.0 (Vienna, Austria; http://www.R-project.org).

### Ethics

This study adhered to the ethical tenets of the Declaration of Helsinki and was approved by the Institutional Review Board of SMC in Seoul, Korea (IRB number: 2022-05-062). The need for informed consent was waived because of the low risk posed by this investigation.

### Reporting summary

Further information on research design is available in the Nature Research Reporting Summary linked to this article.

### Supplementary information

supplemental table S1

REPORTING SUMMARY

## Data Availability

The data sets generated during and/or analyzed during the present study are available from the corresponding author on reasonable request. Reference sequences for *BRCA1* and *BRCA2* DNA numbering were GenBank accession numbers NM_007294.2 and NM_000059.3, respectively^16^.
