# Supplementary material for: Long-term oncologic outcomes of unselected triple-negative breast cancer patients according to BRCA1/2 mutations
Source: NPJ Precis Oncol. 2024 Apr 30;8:96. doi: 10.1038/s41698-024-00559-0 (PMC11061194; doi:10.1038/s41698-024-00559-0)
Supplement: Supplementary file 1 — supplemental table S1 [file 41698_2024_559_MOESM1_ESM.pdf]

Supplemental Table S1. Cox regression model for contralateral breast cancer incidence risk

	Univariate model		Multivariate model	
	HR (95% CI)	P value	HR (95% CI)	P value
Age				
Age<40 vs Age≥40	2.011(0.934-4.327)	0.074	1.663(0.764-3.621)	0.200
pTNM stage				
Stage 2-3 vs Stage 0-1	0.806(0.377-1.726)	0.579	0.771(0.359-1.657)	0.505
Stage pCR vs Stage 0-1	1.2249(0.272-5.508)	0.791	1.246(0.275-5.638)	0.775
BRCA1/2 mutation				
BRCA1/2(+) vs *BRCA1/2*(-)	6.580(3.169-13.664)	<0.0001	6.242(2.985-13.055)	<0.0001

* Reference group for each variable is Age≥40, Stage 0-1, non-*BRCA1/2*
