# Supplementary material for: Long-term oncologic outcomes of unselected triple-negative breast cancer patients according to BRCA1/2 mutations
Source: NPJ Precis Oncol. 2024 Apr 30;8:96. doi: 10.1038/s41698-024-00559-0 (PMC11061194; doi:10.1038/s41698-024-00559-0)
Supplement: Supplementary file 2 — REPORTING SUMMARY [file 41698_2024_559_MOESM2_ESM.pdf]

nature portfolio

Corresponding author(s): DAPR NPJPRECISIONONCOLOGY-04150R2

Last updated by author(s): Feb 18, 2024

Reporting Summary

Nature Portfolio wishes to improve the reproducibility of the work that we publish. This form provides structure for consistency and transparency in reporting. For further information on Nature Portfolio policies, see our Editorial Policies and the Editorial Policy Checklist.

Statistics

For all statistical analyses, confirm that the following items are present in the figure legend, table legend, main text, or Methods section.

n/a	Confirmed	
☐	☒	The exact sample size (*n*) for each experimental group/condition, given as a discrete number and unit of measurement
☐	☒	A statement on whether measurements were taken from distinct samples or whether the same sample was measured repeatedly
☐	☒	The statistical test(s) used AND whether they are one- or two-sided<br>*Only common tests should be described solely by name; describe more complex techniques in the Methods section.*
☐	☒	A description of all covariates tested
☐	☒	A description of any assumptions or corrections, such as tests of normality and adjustment for multiple comparisons
☐	☒	A full description of the statistical parameters including central tendency (e.g. means) or other basic estimates (e.g. regression coefficient) AND variation (e.g. standard deviation) or associated estimates of uncertainty (e.g. confidence intervals)
☐	☒	For null hypothesis testing, the test statistic (e.g. *F*, *t*, *r*) with confidence intervals, effect sizes, degrees of freedom and *P* value noted<br>*Give P values as exact values whenever suitable.*
☐	☒	For Bayesian analysis, information on the choice of priors and Markov chain Monte Carlo settings
☐	☒	For hierarchical and complex designs, identification of the appropriate level for tests and full reporting of outcomes
☐	☒	Estimates of effect sizes (e.g. Cohen's *d*, Pearson's *r*), indicating how they were calculated

Our web collection on statistics for biologists contains articles on many of the points above.

Software and code

Policy information about availability of computer code

Data collection	no software was used
Data analysis	SAS version 9.4 (SAS Institute, Cary, NC, USA) and R3.4.0 (Vienna, Austria; http://www.R-project.org)

For manuscripts utilizing custom algorithms or software that are central to the research but not yet described in published literature, software must be made available to editors and reviewers. We strongly encourage code deposition in a community repository (e.g. GitHub). See the Nature Portfolio guidelines for submitting code & software for further information.

Data

Policy information about availability of data

All manuscripts must include a data availability statement. This statement should provide the following information, where applicable:

- Accession codes, unique identifiers, or web links for publicly available datasets
- A description of any restrictions on data availability
- For clinical datasets or third party data, please ensure that the statement adheres to our policy

The data sets generated during and/or analyzed during the present study are available from the corresponding author on reasonable request. Reference sequences for BRCA1 and BRCA2 DNA numbering were GenBank accession numbers NM_007294.2 and NM_000059.3, respectively

Research involving human participants, their data, or biological material

Policy information about studies with human participants or human data. See also policy information about sex, gender (identity/presentation), and sexual orientation and race, ethnicity and racism.

Reporting on sex and gender	This information has not been collected
Reporting on race, ethnicity, or other socially relevant groupings	Race, ethinicity or other socially relevant grouping were not used or analyzed in our study
Population characteristics	953 non-metastatic triple negative breast cancer patients with samples available for BRCA1/2 genetic testing, who received surgery in our institution from June, 2008 to January, 2016
Recruitment	This was a non-randomized retrospective study it included incomplete patient data, which introduces the possibility of other confounding variables, whether measured or unmeasured. since the collected data is relatively old, there may be unclear or missing information that may have weakened or strengthened the results.
Ethics oversight	Institutional Review Board of Samsung Medical Center, Seoul, Korea

Note that full information on the approval of the study protocol must also be provided in the manuscript.

Field-specific reporting

Please select the one below that is the best fit for your research. If you are not sure, read the appropriate sections before making your selection.

☒ Life sciences ☐ Behavioural & social sciences ☐ Ecological, evolutionary & environmental sciences

For a reference copy of the document with all sections, see nature.com/documents/nr-reporting-summary-flat.pdf

Life sciences study design

All studies must disclose on these points even when the disclosure is negative.

Sample size	Study population was based on samples available for BRCA1/2 genetic testing, with sufficient overall follow-up period. Triple negative breast cancer proportion is approximately 10%-20% of all breast cancer subtypes. We can estimate that this study analyzed the TNBC patients of approximately 5,000-10,000 all breast cancer patients in Korea, which we thought that the significant results of this study are of meaningful value.
Data exclusions	When analyzing the contralateral breast cancer incidence, patients initially diagnosed with bilateral breast cancer were excluded from the analysis. In patients initially diagnosed with bilateral breast cancer, it is impossible to define whether the recurred breast cancer is contralateral or ipsilateral. Therefore, we exlcuded these patients when analyzing contralateral breast incidence.
Replication	This study was a retrospective study analyzing data from retrospective chart review, not an experimental research. The datasets generated during and/or analyzed during the present study are available from the corresponding author on reasonable request. If the same data is used, our results are reproducible.
Randomization	Due to the non-randomization retrospective nature of this study, randomization was not performed. Our results compared the outcomes of triple negative breast cancer patients according to BRCA1/2 mutation status.
Blinding	Due to the non-randomization retrospective nature of this study, blinding was not possible.

Reporting for specific materials, systems and methods

We require information from authors about some types of materials, experimental systems and methods used in many studies. Here, indicate whether each material, system or method listed is relevant to your study. If you are not sure if a list item applies to your research, read the appropriate section before selecting a response.

Materials & experimental systems

n/a	Involved in the study
☒	☐ Antibodies
☒	☐ Eukaryotic cell lines
☒	☐ Palaeontology and archaeology
☒	☐ Animals and other organisms
☒	☐ Clinical data
☒	☐ Dual use research of concern
☒	☐ Plants

Methods

n/a	Involved in the study
☒	☐ ChIP-seq
☒	☐ Flow cytometry
☒	☐ MRI-based neuroimaging

Plants

Seed stocks	*Report on the source of all seed stocks or other plant material used. If applicable, state the seed stock centre and catalogue number. If plant specimens were collected from the field, describe the collection location, date and sampling procedures.*
Novel plant genotypes	*Describe the methods by which all novel plant genotypes were produced. This includes those generated by transgenic approaches, gene editing, chemical/radiation-based mutagenesis and hybridization. For transgenic lines, describe the transformation method, the number of independent lines analyzed and the generation upon which experiments were performed. For gene-edited lines, describe the editor used, the endogenous sequence targeted for editing, the targeting guide RNA sequence (if applicable) and how the editor was applied.*
Authentication	*Describe any authentication procedures for each seed stock used or novel genotype generated. Describe any experiments used to assess the effect of a mutation and, where applicable, how potential secondary effects (e.g. second site T-DNA insertions, mosiacism, off-target gene editing) were examined.*
